# Artificial Intelligence in *ALK*-Rearranged NSCLC: Forecasting Response and Resistance

**DOI:** 10.3390/cancers18060973

**Published:** 2026-03-18

**Authors:** Andreas Koulouris, Christos Tsagkaris, Konstantinos Kalaitzidis, Georgios Tsakonas, Giannis Mountzios

**Affiliations:** 1 Thoracic Oncology Center, Karolinska University Hospital, 171 76 Stockholm, Sweden; 2 Department of Oncology-Pathology, Karolinska Institutet, 171 77 Stockholm, Sweden; 3 Faculty of Medicine, Aristotle University of Thessaloniki, 54124 Thessaloniki, Greece; 4 Science for Life Laboratory, Department of Biochemistry and Biophysics, Stockholm University, 171 21 Solna, Sweden; 5 Department of Medical Oncology, Bank of Cyprus Oncology Center, Nicosia 28551, Cyprus; 6 Fourth Department of Medical Oncology and Clinical Trials Unit, Henry Dunant Hospital Center, 11526 Athens, Greece

**Keywords:** *ALK* rearrangement, NSCLC, artificial intelligence, machine learning, radiomics, digital pathology, prognostic modeling, treatment response, resistance mechanisms, systematic review

## Abstract

Background/Objectives: The management and prognosis of *ALK*-rearranged non-small-cell lung cancer have substantially improved over the past decade. However, challenges remain in timely molecular identification, prediction of treatment response, and understanding resistance mechanisms. This systematic review evaluates and synthesizes the evidence on artificial intelligence (AI) approaches leveraging imaging, pathology, molecular, and clinical data in this setting. Methods: A systematic search was conducted for peer-reviewed studies published between 2020 and 2025. Eligible studies involved human subjects and applied AI, machine learning, or deep learning methods to predict *ALK* status or treatment-related outcomes using imaging, pathology, molecular, or multimodal data. Study selection followed the PRISMA 2020 guidelines. Data were extracted on study design, data modality, AI methodology, clinical objectives, and performance metrics. Bibliometric co-occurrence analysis was performed to characterize thematic patterns and temporal trends. Results: Thirteen studies met the inclusion criteria, most of which were retrospective and single-center. AI approaches were applied to radiologic, pathologic, molecular, or multimodal data. Models predicting *ALK* status reported area under the curve values ranging from 0.73 to 0.99, while prognostic and treatment-response models reported moderate to high discriminative performance. Bibliometric analysis identified two dominant research themes focused on molecular characterization and computational methodology, with a recent shift toward treatment-specific and integrative analyses. External validation and clinical implementation remained limited across studies. Conclusions: AI shows promising potential to support diagnosis, prognostication, and treatment assessment in *ALK*-rearranged lung cancer. However, methodological heterogeneity, limited external validation, and a lack of prospective studies currently constrain clinical translation.

## Simple Summary

*ALK*-positive non-small-cell lung cancer is a distinct molecular subtype for which targeted therapies have significantly enhanced patient outcomes. However, prediction of treatment response and understanding of resistance mechanisms remain clinically challenging. Artificial intelligence has been increasingly investigated as a tool to support these tasks by analyzing clinical data, imaging, pathology, and molecular features. In this systematic review, we summarize and critically appraise studies applying artificial intelligence to *ALK*-rearranged lung cancer, with a focus on diagnostic, prognostic, and treatment-related applications. We further explore methodological trends and research focus within the field. While many studies report promising predictive performance, most rely on retrospective, single-center data and lack external validation. This review highlights both the potential and current limitations of artificial intelligence in this setting and outlines key requirements for future clinically translatable research.

## 1. Introduction

Lung cancer remains the leading cause of cancer-related mortality worldwide, with non-small-cell lung cancer (NSCLC) accounting for 80–90% of all cases [1]. Advances in molecular profiling have reshaped the management of NSCLC by enabling biomarker-driven treatment strategies. Among actionable oncogenic drivers, rearrangements of the anaplastic lymphoma kinase (*ALK*) gene define a distinct molecular subtype, typically associated with younger age, adenocarcinoma histology, and limited smoking exposure [2]. The development of first-, second-, and third-generation *ALK* tyrosine kinase inhibitors (TKIs) has substantially improved clinical outcomes, transforming *ALK*-rearranged NSCLC into a paradigm of precision oncology [3,4,5,6,7,8,9].

Despite these therapeutic advances, several clinically relevant challenges persist. Accurate and timely identification of *ALK* rearrangements remains essential but can be limited by tissue availability, cost, and turnaround time of molecular testing. Furthermore, heterogeneity in treatment response and the development of both primary and acquired resistance complicate long-term disease control [10,11,12]. These challenges have driven interest in complementary approaches that can enhance molecular characterization, predict therapeutic outcomes, and provide insights into resistance mechanisms beyond conventional diagnostics.

In parallel, artificial intelligence (AI), encompassing machine learning (ML) and deep learning (DL) methodologies, has emerged as a powerful analytical framework in oncology. AI-based models are increasingly applied to complex, high-dimensional datasets derived from radiologic imaging, digital pathology, cytology, molecular profiling, and electronic health records. In NSCLC, such approaches have been explored for nodule characterization, mutation prediction, prognostication, and treatment-response assessment [13,14,15,16]. Recent studies have reported a rapid expansion of clinical prediction models incorporating AI, particularly those leveraging multimodal data to support individualized decision-making across early and metastatic disease settings [15,16,17,18,19].

Within this broader landscape, AI applications targeting molecularly defined subgroups of NSCLC are of particular interest. Several original studies have demonstrated the feasibility of predicting *ALK* status directly from computed tomography (CT), positron emission tomography/computed tomography (PET-CT), histopathology slides, or cytologic specimens using ML and DL techniques [14,20,21,22]. Beyond molecular classification, AI-driven models have been developed to estimate prognosis, predict response to *ALK* TKIs, and explore treatment resistance through radiomics, bioinformatic analyses, and molecular modeling [10,11,12,17,23]. These approaches offer the potential to complement standard molecular testing, support treatment selection, and enhance understanding of disease biology in *ALK*-rearranged NSCLC.

However, despite growing interest and promising performance metrics, the current evidence base remains fragmented. Existing reviews addressing AI in NSCLC often focus on heterogeneous patient populations, broad biomarker groups, or general clinical prediction models, limiting *ALK*-specific insights [11,16,19]. Moreover, concerns regarding methodological heterogeneity, limited external validation, and the predominance of retrospective, single-center studies continue to restrict clinical translation of AI-based tools [11,12,16,19,24].

Consequently, a focused synthesis of AI applications specifically applied to *ALK*-rearranged NSCLC is warranted. The present systematic review aims to evaluate and synthesize the published evidence on AI-based approaches for molecular prediction, prognostication, treatment-response assessment, and mechanistic exploration in *ALK*-rearranged NSCLC. By integrating a systematic literature review conducted in accordance with PRISMA 2020 guidelines with bibliometric co-occurrence analysis, this study seeks to characterize research trends, dominant thematic areas, and existing gaps, thereby informing future research priorities and supporting the development of clinically meaningful AI-driven strategies in this molecularly defined subset of lung cancer [25,26].

## 2. Materials and Methods

### 2.1. Literature Search Strategy

A systematic search of peer-reviewed literature was conducted in PubMed/MEDLINE and Google Scholar to synthesize and critically appraise AI-based approaches applied to *ALK*-rearranged NSCLC. The search covered articles published in English between 1 January 2020 and 1 January 2026, and the final search was performed on 1 January 2026. Search terms combined keywords related to *ALK*-rearranged NSCLC with AI methodologies and clinically relevant outcomes, including molecular diagnosis, treatment response, resistance, prognosis, and survival. The following Boolean search strategy was applied: (“*ALK*” OR “anaplastic lymphoma kinase”) AND (“non-small cell lung cancer” OR “NSCLC”) AND (“artificial intelligence” OR “machine learning” OR “deep learning” OR “radiomics” OR “pathomics” OR “multiomics”). Google Scholar yielded approximately 15,700 results using the same search terms during the same time period. Due to the large number of results retrieved, screening was limited to the first ten pages of results (100 records) sorted by relevance, following commonly used approaches in systematic reviews. No field restrictions (e.g., title/abstract filters) were applied to maximize search sensitivity. Reference lists of eligible articles were also screened to identify additional relevant studies.

### 2.2. Eligibility Criteria

Studies were eligible for inclusion if they (a) involved human subjects or computational/translational analyses relevant to *ALK*-rearranged NSCLC; (b) reported original research; (c) applied AI, ML, or DL techniques to imaging, pathology, molecular, clinical, or multimodal data; and (d) addressed *ALK* rearrangement detection or treatment-related outcomes in *ALK*-positive NSCLC. Exclusion criteria included review articles, editorials, conference abstracts, studies without *ALK*-specific analyses, absence of reported performance metrics—e.g., area under the curve (AUC), sensitivity, specificity, C-index—and unavailability of full-text articles in English. Given the exploratory nature of AI applications in *ALK*-rearranged NSCLC, translational studies employing in silico modeling or AI-driven drug discovery approaches were also considered eligible when they directly addressed *ALK* biology, therapeutic response, or resistance mechanisms.

### 2.3. Study Selection and Data Extraction

Study selection was performed in accordance with the Preferred Reporting Items for Systematic Reviews and Meta-Analyses (PRISMA) guidelines [25]. Titles and abstracts were screened for relevance, followed by full-text assessment of potentially eligible studies. Screening and data extraction were conducted independently by two reviewers, with discrepancies resolved by consensus. Data extracted from included studies comprised publication year, geographic origin, study design, sample size, data modality, artificial intelligence methodology, clinical objectives, validation strategy, and reported performance metrics. When study-level variables were missing or unclearly reported, no assumptions were made and the information was recorded as not available. Outcomes of interest included diagnostic performance for ALK rearrangement detection, prognostic outcomes, such as progression-free survival (PFS) and overall survival (OS), treatment-response measures to ALK TKIs, and mechanistic or resistance-related molecular findings. All reported results relevant to these outcome domains were collected. The completed PRISMA 2020 checklist is provided in Supplementary Material. This review was not prospectively registered, because it was conceived as an exploratory synthesis of a rapidly evolving and methodologically heterogeneous field. Consequently, no formal review protocol was publicly deposited prior to study initiation.

### 2.4. Risk of Bias Assessment

A formal risk-of-bias assessment using a standardized tool could not be uniformly applied across all study types, as no validated instrument currently exists that adequately captures the methodological heterogeneity of AI-based prediction studies spanning imaging, pathology, molecular, multimodal analyses, and in silico designs. Instead, a structured qualitative risk-of-bias assessment was performed based on methodological domains commonly considered in AI prediction research and inspired by established frameworks such as QUADAS-2 and PROBAST [27,28,29]. The assessment focused on four domains: (a) patient selection and dataset representativeness, (b) risk of data leakage and model overfitting, (c) validation strategy (internal versus external), and (d) model calibration and clinical applicability. Each included study was qualitatively evaluated across these domains based on the information reported in the original publications. The outcomes of this assessment are further discussed in Section 3 and Section 4.

### 2.5. Bibliometric and Thematic Analysis

To explore thematic patterns within the included literature, a bibliometric co-occurrence analysis was performed using VOSviewer, version 1.6.20 (Leiden University, Leiden, The Netherlands) [26]. Terms were extracted from titles and abstracts, and those occurring at least three times were retained using the default relevance threshold of 80%. Manual screening was undertaken to remove syntactic or non-contextual terms. Network and temporal overlay visualizations were subsequently generated to characterize conceptual clustering, thematic relationships, and temporal evolution in research focus across the included studies.

### 2.6. AI-Assisted Language Editing

ChatGPT (version 5.2, OpenAI) was used solely for language editing and proofreading. The authors reviewed and verified all AI-assisted modifications and take full responsibility for the content of the manuscript.

## 3. Results

### 3.1. Overview

**Figure 1:**
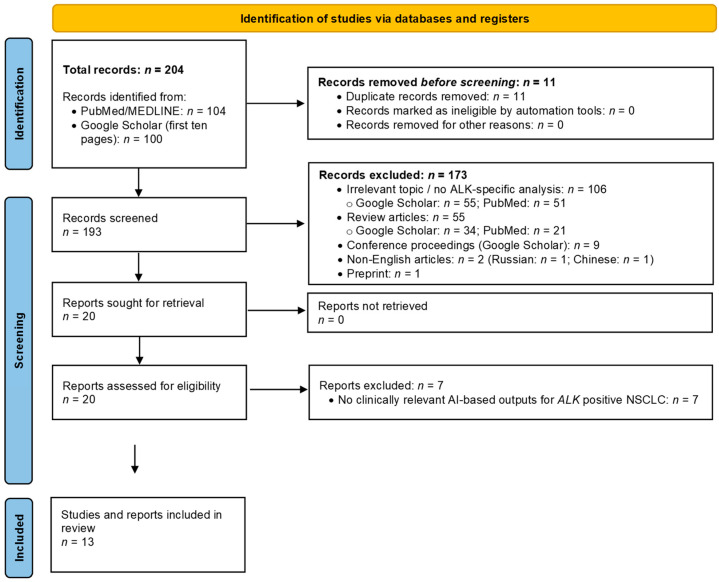
PRISMA 2020 flow diagram of study identification, screening, eligibility, and inclusion.

A total of 204 records were identified through database and search engine screening, of which 11 duplicates were removed prior to screening. Following title and abstract screening, 20 studies were deemed eligible for full-text assessment, of which 13 met the predefined inclusion criteria and were included in the final analysis (Figure 1 and Table 1). The excluded studies were primarily reviews, conference proceedings, methodological papers without *ALK*-specific analyses, or investigations lacking clinically relevant AI outputs. The included studies were published between 2020 and 2025 and were predominantly retrospective in design.

#### 3.1.1. Geographic Distribution

The majority of studies originated from East Asia, particularly China and Japan, with additional contributions from Europe and Israel. Sample sizes varied widely, ranging from small exploratory cohorts to large population-based datasets exceeding 5000 individuals. Most investigations were single-center studies.

#### 3.1.2. Focus Areas, Data, and Analytical Approaches

The included studies retrieved data from imaging, pathology, and clinical records and used a wealth of AI modalities to analyze them. Imaging-based approaches constituted the largest group, predominantly using CT or PET/CT data to extract radiomic features for model training and prediction of *ALK* rearrangement or clinical outcomes [12,22,23,31]. The second largest group relied on pathology or cytology, applying convolutional neural networks to whole-slide images or cytological specimens for the prediction of genetic alterations, including *ALK* and *ROS1* fusions [11,14,19,24]. A smaller number of investigations incorporated multimodal data, combining radiologic, pathological, and clinical records [12,21,23]. Finally, a case report used post hoc molecular modeling and an in silico study harnessed mutation database analyses to identify molecular traits associated with resistance to TKIs [10,22].

AI methodologies ranged from conventional ML approaches, including logistic regression, random forest models, and least absolute shrinkage and selection operator (LASSO) regression, to DL architectures, such as convolutional neural networks and ensemble learning models [11,14,23]. In silico studies applying AI to molecular modeling and drug discovery formed a distinct subgroup, focusing on the identification of candidate *ALK* inhibitors or the mechanistic characterization of resistance-associated alterations, including rare fusion variants with a potential to alter molecular docking and subsequent outcomes associated with TKI administration [10,20,30].

#### 3.1.3. Prediction Goals and Clinical Objectives

Clinical objectives identified in the included studies can be grouped under three categories: (a) *ALK* status, (b) prognostic or predictive modeling in *ALK*-positive NSCLC, and (c) molecular/genetic characterization. *ALK* status was predicted from imaging or pathological data. These studies primarily used radiomics or DL-based approaches reporting AUC values ranging from approximately 0.73 to 0.99 depending on data modality and model architecture [11,14,23,24].

Prognostic or predictive modeling in *ALK*-positive NSCLC was designed to estimate PFS, OS, or response to *ALK* TKIs. Radiomics-based and multimodal approaches reported moderate-to-high discriminative performance, with reported concordance indices ranging from 0.72 to 0.89 and significant associations with clinical outcomes [12,30,31].

Studies exploring mechanistic or translational applications of AI focused on the identification of resistance-associated molecular signatures and in silico screening of candidate *ALK* inhibitors. Insights were provided into structure–function relationships and resistance mechanisms in the context of either identifying genes to be targeted in the frame of drug discovery or post hoc identifying genes or molecular folding traits associated with resistance to TKIs [10,20,22].

#### 3.1.4. Outcome and AI Performance Metrics

Model performance was reported using AUC, sensitivity, specificity, and C-index values. For studies addressing *ALK* rearrangement detection, AUC values typically ranged between 0.73 and 0.99, with higher discriminative performance generally observed in pathology-based and multimodal models, although these estimates should be interpreted cautiously given differences in dataset composition, validation strategies, and disease prevalence [11,14,24]. Prognostic models demonstrated moderate discriminative ability, with C-index values slightly over 0.70 in both training and validation cohorts [30,31]. External validation was reported in only a minority of studies, while most models were evaluated using internal cross-validation or single-cohort testing. Finally, although one study employed a prospective observational design, none of the included investigations assessed AI models in a prospective clinical decision-making or implementation setting [13].

#### 3.1.5. Risk of Bias Assessment

A structured qualitative risk-of-bias assessment was conducted across four methodological domains, and the results are summarized in Table 2. Most included studies were retrospective and single-center, which may limit dataset representativeness and generalizability. Sample sizes varied substantially across studies, ranging from small exploratory cohorts to large registry-based datasets. The risk of model overfitting was considered moderate in most studies, but it was more pronounced in smaller cohorts. The majority of studies relied on internal validation approaches, and calibration metrics were infrequently reported. Moreover, clinical applicability varied across study types. Diagnostic and prognostic prediction models demonstrated potential translational relevance, whereas in silico modeling studies and the single-patient case report were considered exploratory and hypothesis-generating.

### 3.2. Bibliometry

**Figure 2:**
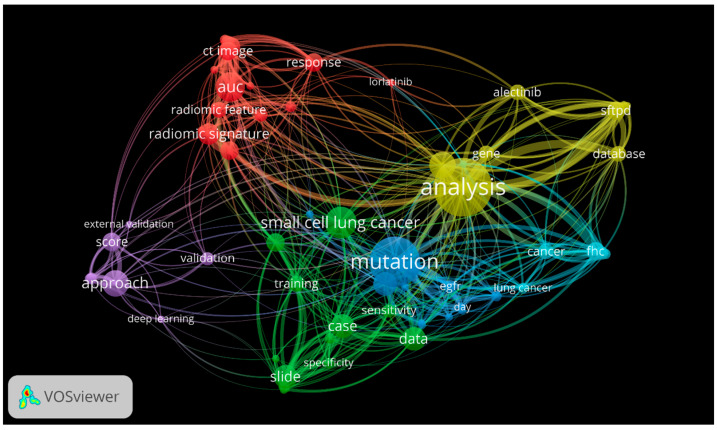
Network visualization showing keyword frequency and co-occurrence across the included studies. Generated using VOSviewer, version 1.6.20 (Leiden University, The Netherlands).

An overview of thematic networks based on frequency and co-occurrence is presented in Figure 2. The visualization demonstrated two dominant conceptual groups, “mutation” and “analysis”, indicating a molecular characterization and computational methodology anchor over clinical decision-making. Radiomics-related terms (e.g., AUC, CT image, radiomic signature) formed a key analysis cluster, reflecting the focus on imaging-based AI models and their performance evaluation. A second major cluster was centered on mutation, gene, and targeted therapies (including alectinib and lorlatinib), representing biologically informed and treatment-relevant analyses. Digital-pathology-related terms (e.g., slide, data) constituted a smaller but distinct cluster, indicating transversal use of methodologies across studies in the field.

**Figure 3:**
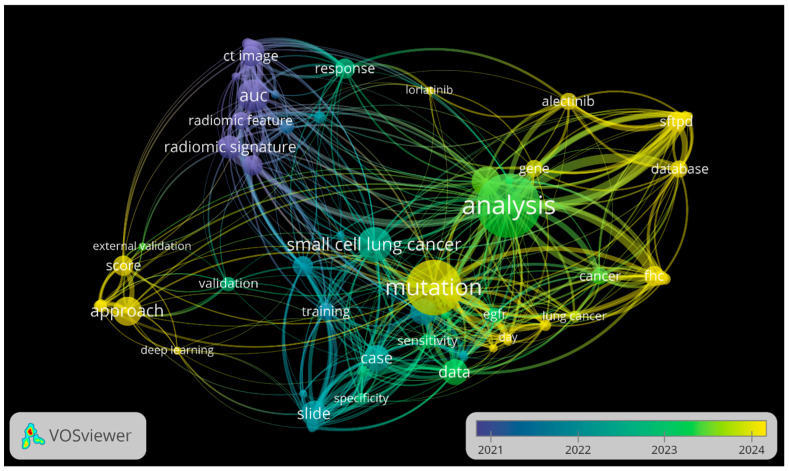
Temporal overlay visualization of keyword frequency and co-occurrence. Generated using VOSviewer, version 1.6.20 (Leiden University, The Netherlands).

Temporal overlay analysis demonstrated an evolution pattern in research questions, as presented in Figure 3. Studies published between 2021 and 2022 were predominantly centered on radiomic performance metrics and feasibility assessments. In contrast, publications from 2023–2024 increasingly emphasized molecular analysis, treatment-specific contexts, and integrative modeling approaches, suggesting a gradual transition from methodological proof-of-concept toward clinically oriented applications. Notably, terms related to validation and clinical implementation remained peripheral throughout the network.

## 4. Discussion

This systematic review synthesizes current evidence on AI-based applications in NSCLC with *ALK* rearrangement, highlighting both the promise and the limitations of this rapidly evolving landscape. Across thirteen eligible studies published between 2020 and 2025, AI was applied to imaging, pathology, molecular datasets, and multimodal clinical data to support molecular identification, prognostication, treatment-response assessment, and mechanistic exploration. Collectively, the findings suggest that AI-based approaches can achieve moderate-to-high discriminative performance in selected tasks, particularly for *ALK* rearrangement detection, while underscoring substantial gaps in validation, clinical integration, and prospective evaluation.

A major finding of this review is the relatively consistent performance of AI models designed to predict *ALK* status directly from imaging or histopathological data. Reported AUC values ranging from 0.73 to 0.99 indicate that both radiomics-based and digital pathology approaches can capture phenotypic correlates of *ALK*-driven disease. Pathology-based DL models, particularly those applied to hematoxylin-eosin-stained slides or cytologic specimens, demonstrated the highest discriminative performance, in some cases approaching near-perfect classification accuracy. These findings align with broader observations in NSCLC that digital pathology-based AI models outperform imaging-only approaches for the prediction of genetic alterations [12,16,19].

Despite promising performance metrics, these models were predominantly developed and tested in retrospective, single-center cohorts. As illustrated in other recent reviews regarding clinical prediction models in NSCLC, favorable internal performance does not necessarily translate into real-world clinical utility, particularly in the absence of external validation and standardized workflows [16]. In the *ALK*-specific context, this limitation is of particular importance, as even modest reductions in specificity could have significant clinical implications given the low prevalence of *ALK* rearrangements in unselected NSCLC populations [1,2].

Beyond molecular classification, a subset of included studies focused on prognostic modeling and treatment-response prediction in *ALK*-positive NSCLC. Radiomics-based and multimodal models demonstrated moderate-to-high discriminative ability for PFS and OS, with C-index values generally exceeding 0.70. These findings align with broader NSCLC AI literature, indicating that radiomics and clinical–radiologic models can provide complementary prognostic information, while multimodal approaches integrating imaging with pathology and clinical variables are increasingly emphasized for risk stratification and treatment outcome prediction [15,18,32].

Remarkably, the inclusion of *ALK*-positive cohorts in prognostic AI studies remains limited, and most models were not specifically designed to guide therapeutic strategies among different *ALK* TKIs. This contrasts with emerging AI-based clinical prediction models in unselected NSCLC populations, which increasingly incorporate treatment-specific variables and attempt to predict outcomes under distinct therapeutic strategies [16]. In unselected NSCLC, DL survival models incorporating tumor stage, clinical features, and treatment modalities have demonstrated superior prognostic performance in comparison with conventional staging systems, and have even been explored as tools to support individualized treatment recommendations [33,34].

In *EGFR*-mutant NSCLC, AI-driven prognostic models have progressed beyond risk stratification to treatment-response prediction. DL frameworks integrating imaging-derived semantic features have been shown to identify patients unlikely to benefit from *EGFR*-TKIs, with external validation across multiple cohorts and direct implications for therapeutic selection [35]. These developments highlight how AI can be leveraged not only for prognosis but also to inform precision treatment strategies in oncogene-driven NSCLC.

In early-stage NSCLC, multiple ML-based prognostic models have been developed using radiomics, digital pathology, and clinicopathological variables to predict OS or recurrence risk, often with multicenter validation. These models aim to identify patients who may benefit from adjuvant therapy beyond standard staging criteria [36,37]. However, none of these approaches have focused specifically on *ALK*-rearranged tumors, despite their distinct biology and therapeutic trajectories.

The relative scarcity of *ALK*-specific prognostic and predictive AI models, therefore, represents a critical gap. Given the evolving landscape of *ALK* inhibitors and the heterogeneous patterns of resistance, AI tools tailored to *ALK*-rearranged NSCLC could play a crucial role in optimizing treatment sequencing, anticipating resistance, and refining long-term disease management. The contrast between the maturity of AI-based prognostic modeling in *EGFR*-mutant or unselected NSCLC and its limited application in *ALK*-positive disease underscores an unmet need for *ALK*-focused predictive frameworks.

An important, albeit smaller, body of work has explored the application of AI to the investigation of resistance mechanisms to *ALK* TKIs. In silico studies leveraging molecular modeling, mutation databases, and bioinformatic analyses identified candidate resistance-associated genes, signaling pathways, and structural alterations affecting *ALK* inhibitor binding. These studies provide biologically plausible insights into resistance mechanisms, including rare fusion variants and conformational changes that may underlie poor responses to specific *ALK* inhibitors [14,31]. Nevertheless, the biological relevance of these computational predictions ultimately requires validation through functional assays and genomic analyses in experimental and clinical datasets. While such approaches align with broader trends in AI-driven molecular oncology, their clinical relevance remains largely hypothetical, as most mechanistic AI studies often lack direct linkage to patient-level outcomes or prospective validation [32]. As a result, their contribution currently lies more in hypothesis generation and drug discovery than in immediate clinical decision support.

The bibliometric mapping performed in this review revealed two key tendencies in the current literature on AI in *ALK*-rearranged NSCLC. The body of research is primarily organized around methodology rather than clinical decision-making pathways. Temporal overlay analysis demonstrated a shift from feasibility studies focused on imaging-derived performance metrics in 2021–2022 toward more recent studies integrating clinical, imaging, pathological, and molecular features for prediction and prognostication, reflecting a gradual maturation of the field. However, aspects concerning validation, clinical implementation, and real-world applicability remained peripheral, underscoring a persistent gap between model development and translational utility [16]. In the *ALK*-specific context, this gap may be further exacerbated by small cohort sizes, molecular heterogeneity, and rapidly evolving treatment paradigms. Accordingly, the bibliometric patterns identified in this review should be interpreted as exploratory, reinforcing the need for future research to move beyond performance benchmarking toward prospectively validated, clinically integrated AI frameworks.

Several limitations should be considered when interpreting the findings of this review. The number of eligible studies focusing specifically on *ALK*-rearranged NSCLC remains small, which restricts the generalizability of conclusions. Most included studies were retrospective and single-center, with heterogeneous datasets, feature selection strategies, AI methodologies, and reported performance metrics, precluding quantitative synthesis or meta-analysis. Only one study employed a prospective observational design; however, even this investigation did not assess AI models in prospective clinical deployment or real-time decision-support integration. External validation was reported infrequently, with most models relying on internal cross-validation or single-cohort testing, raising concerns regarding overfitting and real-world applicability.

Furthermore, performance metrics, such as AUC and C-index, should be interpreted with caution. In the context of ALK-rearranged NSCLC, the relatively low prevalence of ALK alterations in unselected NSCLC populations may substantially affect the positive predictive value of AI-based prediction models, even when discrimination metrics appear favorable [2]. Consequently, models demonstrating high AUC values in retrospective datasets may yield limited clinical utility when applied to broader patient populations. Notably, most studies did not report threshold selection strategies, calibration metrics, or decision-curve analyses, which are essential for evaluating the clinical usefulness of prediction models and their impact on real-world decision-making. Without these assessments, it remains difficult to determine how model predictions would translate into clinically actionable thresholds or treatment decisions. A formal risk-of-bias assessment using a standardized tool was not feasible due to the methodological heterogeneity of AI-based studies. Instead, a structured qualitative assessment across key methodological domains was performed, although differences in study design and their inherent limitations still constrain the strength of comparative inferences. Finally, publication bias and selective reporting of high-performing models may have contributed to the overestimation of real-world performance.

Future research in this field should prioritize methodological rigor and clinical relevance. Multi-center prospective studies with standardized data acquisition, transparent reporting, and robust external validation are essential to establish the generalizability of AI models across imaging, pathology, and molecular platforms. Emerging collaborative approaches, such as federated learning, which enable model training across multiple institutions without direct data sharing, may help overcome data privacy constraints and mitigate single-center biases while facilitating the development of more generalizable AI models [38].

In *ALK*-rearranged NSCLC, there remains a clear unmet need for AI frameworks capable of integrating longitudinal clinical and molecular data to predict response and resistance to successive *ALK* inhibitors. In clinical practice, AI-based models may function as triage tools, identifying patients with a high probability of *ALK* rearrangements in tissue-limited cases, thereby potentially optimizing diagnostic workflows, guiding the need for re-biopsy, and reducing delays in targeted therapy initiation. AI may also contribute to rational drug development and resistance mitigation. In *EGFR*-mutated NSCLC, computational modeling and high-throughput structure–activity relationship (SAR) analyses, enhanced by AI, may inform next-generation inhibitor design and accelerate precision drug discovery [39]. Similar AI-assisted SAR-driven strategies could be extended to *ALK* biology, particularly to address resistance-conferring variants and conformational alterations identified through in silico modeling studies.

Another promising avenue lies in the integration of AI with established prognostic frameworks for brain metastases, a major clinical challenge in *ALK*-rearranged NSCLC. Prognostic tools, such as the Lung-molGPA and the *ALK*-Brain Prognostic Index (*ALK*-BPI), have demonstrated clinical utility and external validation in stratifying survival risk [40,41,42]. AI-driven augmentation of these prognostic scores could enable more individualized prognostication and treatment planning for these patients.

In addition, AI-enhanced liquid biopsy approaches represent a rapidly evolving frontier with particular relevance for *ALK*-positive disease. Integration of ML with circulating tumor DNA (ctDNA) analysis has shown potential to improve mutation detection sensitivity, identify minimal residual disease, and detect relapse earlier than conventional imaging [43,44]. In the context of *ALK*-rearranged NSCLC, such approaches could support non-invasive monitoring of resistance mutations, guide treatment switching, and complement radiologic and tissue-based assessments within multimodal AI frameworks.

Finally, future multimodal AI systems may benefit from emerging DL architectures capable of integrating heterogeneous clinical data streams, including imaging, pathology, genomic profiles, and electronic health records within unified analytical frameworks. Such models could function as clinical decision-support systems embedded in routine workflows, assisting physicians in interpreting complex multimodal datasets and guiding individualized treatment strategies [45,46]. Adaptive AI systems incorporating longitudinal data, such as serial imaging, biomarker dynamics, or ctDNA levels, may enable real-time monitoring of treatment response and earlier detection of therapeutic resistance, thereby paving the way for more responsive and personalized disease management [43,45].

## 5. Conclusions

AI applications in *ALK*-rearranged NSCLC show promising potential across molecular prediction, prognostication, treatment-response assessment, and exploratory analysis of resistance mechanisms using imaging, pathology, molecular, and multimodal data. However, their evidence is constrained by small study numbers, methodological heterogeneity, limited external validation, and a lack of prospective clinical implementation. Future advances will depend not only on improved model performance but also on the thoughtful integration of AI into clinically meaningful pathways. Bridging the gap between algorithmic innovation and real-world clinical impact will require rigorous prospective validation and close multidisciplinary collaboration between clinicians, data scientists, and regulatory stakeholders to ensure safe and effective clinical translation in *ALK*-rearranged NSCLC.

## Figures and Tables

**Table 1:** Overview of the included studies, their methodologies, and outcomes.

Author, Year	Country	Study Type	Data Modality	Sample Size	AI Method	Clinical Aim	*ALK*-Specific Output
Terada et al., 2022 [24]	Japan	Retrospective	Pathology/ IHC	*n* = 208	DL (HALO AI, Dense Net)	*ALK* rearrangement prediction	AUC = 0.73; 73% sensitivity and specificity vs. 13% and 94% human specificity and sensitivity.
Chen et al., 2025 [13]	China	Retrospective	Imaging/ CT	*n* = 250	Deep Wise AI workstation	Prediction of growth rate and heterogeneity in solid NSCLC nodules	AUC = 0.704; sensitivity: 65.5%, specificity: 70.5%; *ALK* rearrangements enriched in high-grade adenocarcinomas, no significant correlation between *ALK* and growth rate.
Trinh et al., 2025 [20]	France	In silico	Pharmacology/Drug Design	*n*= 120,571 compounds	ML (XGBoost algorithm), DL (ANN)	Screening of novel *ALK* inhibitors	EV-f1 score of 0.921, EV-average precision of 0.961, cross-validation-f1 score; 3 promising *ALK* inhibitors identified.
Barberis et al., 2024 [10]	Italy	In silico/ in Vivo	Molecular/Digital Pathology	*n* = 1	AlphaFold2, 3	Molecular modeling to investigate poor clinical response to alectinib	Rare striatin *STRN-ALK* fusion distorting alectinib’s binding pocket.
Calvo et al., 2024 [21]	Spain	Retrospective	AI-assisted statistical analysis of multimodal records (clinical, imaging, genetics)	*n*= 5788 patients, 939 with family history of cancer, 552 with *EGFR* or *HER2* mutation or *ALK* translocation	Knowledge Graph and Unified Schema	Cancer risk estimation	At least one relative with cancer among 9.53% of patients with *ALK* translocation or *EGFR* or *HER2* mutations; *ALK* translocation: the most common among young female non-smokers.
Li et al., 2024 [22]	China	Retrospective	In Silico/Bioinformatics	Not applicable (Gene Encyclopedias/ Databases)	DAVID	Gene and signaling pathways identification in alectinib-resistant NSCLC	Five hub genes identified (*MUC5B, SFTPD, DMBT1, SFTPA2, TFF3*).
Mayer et al., 2022 [14]	Israel	Retrospective	Pathology	*n* = 234	Computer vision, CNN	Identification of *ALK* and *ROS1* fusions in NSCLC	AUC: 1 for *ALK* fusion, 0.93 for *ROS1* fusion, Sensitivity and specificity 100% and 100% for *ALK* fusion, 100% and 98.48% for *ROS1* fusion, respectively.
Ishii et al., 2022 [19]	Japan	Retrospective	Cytology	*n* = 138 (106 with cancer, 32 controls)	ML, MobileNet-V2	Gene alteration prediction model	Accuracy: 0.945; Precision: 0.991.
Tan et al., 2022 [11]	China	Retrospective	Pathology	*n* = 1089	DL, ML, stacked ensemble model	Prediction of *EGFR* mutations (including uncommon mutations) and *ALK* rearrangement	AUC of 0.995 and 0.921 in the training and testing cohorts for *ALK* rearrangement, overall AUC of 0.93 and 0.83 in the training and testing cohorts.
Chang et al., 2021 [23]	China	Retrospective	Radiology (PET/CT) and clinical data	*n* = 526 (109 with and 417 without *ALK* rearrangements)	AI Kit (mRMR and LASSO logistic regression)	*ALK* rearrangement Status	AUC = 0.87 in the training group; AUC = 0.88 in the testing group; Specificity = 0.94, Sensitivity = 0.58.
Song et al., 2021 [12]	China	Retrospective	Radiology, Pathology, Clinical data	*n*= 937	ML, 3 blocks model (CT, clinicopathological classifier)	Predict *ALK* status and response to *ALK*-TKI therapy	AUC = 0.8540 in the primary cohort, AUC 0.8481 in the validation cohort; PFS of 16.8 vs. 7.5 months (*p* = 0.010) for patients predicted as *ALK* (+) and *ALK* (−), respectively.
Li et al., 2020 [30]	China	Retrospective	Radiomics	*n* = 63	ML	Prognosis of stage IV *ALK* (+) NSCLC with CT-Based radiomic signature	C-index: 0.744; time-dependent AUC: 0.895 in the training cohort; C-index: 0.717; time-dependent AUC: 0.824 in the validation cohort; Effective risk-stratification prognosis with HR: 2.181 (*p* < 0.001).
Koyama et al., 2024 [31]	Japan	Retrospective	Radiology, Clinical data	*n*= 459 (training group, *n* = 299; testing group, *n* = 160)	RSF algorithm vs. CPH estimation	Personalized survival prediction in patients with advanced NSCLC	C-index 0.841, superior to CPH model (0.775, *p* < 0.001)

AI: artificial intelligence; ALK: anaplastic lymphoma kinase; IHC: immunohistochemistry; DL: Deep learning; AUC: area under the curve; vs.: versus; CT: computed tomography; NSCLC: non-small-cell lung cancer; ML: machine learning; EV: external validation; ANN: artificial neural network; DAVID: database for annotation, visualization, and integrated discovery; MUC5B: mucin 5B; SFTPD: surfactant protein D; DMBT1: deleted in malignant brain tumors 1; SFTPA2: surfactant protein A2; TFF3: trefoil factor 3; CNN: convolutional neural network; mRMR: maximum relevance minimum redundancy; LASSO: least absolute shrinkage and selection operator; HR: hazard ratio; TKIs: tyrosine kinase inhibitors; PFS: progression-free survival; RSF: random survival forest; CPH: Cox proportional hazard;.

**Table 2:** Qualitative risk-of-bias assessment of included studies.

Study	Patient Selection & Representativeness	Overfitting	Validation Strategy	Clinical Applicability
Terada et al., 2022 [24]	Moderate (retrospective single-center cohort, *n* = 208)	Moderate	Internal	Moderate
Chen et al., 2025 [13]	Moderate (retrospective cohort, *n* = 250)	Moderate	Internal	Limited
Trinh et al., 2025 [20]	N/A (compound dataset)	Low	Internal (Cross-validation)	Preclinical
Barberis et al., 2024 [10]	High (single-patient case report)	N/A	None	Hypothesis- generating
Calvo et al., 2024 [21]	Moderate (retrospective registry dataset). 5788 patients with lung cancer in the AI-assisted analysis; *n* = 116 (2%) with *ALK* translocation	Moderate	Internal	Moderate
Li et al., 2024 [22]	N/A (bioinformatic database analysis)	Low	None	Exploratory
Mayer et al., 2022 [14]	Moderate (retrospective pathology cohort, *n* = 234)	Moderate	Internal	Moderate
Ishii et al., 2022 [19]	Moderate (small retrospective cohort, *n* = 138)	Moderate–High	Internal	Limited
Tan et al., 2022 [11]	Moderate (retrospective cohort, *n* = 1089)	Moderate	Internal	Moderate
Chang et al., 2021 [23]	Moderate (retrospective cohort, *n* = 526)	Moderate	Internal	Moderate
Song et al., 2021 [12]	Moderate (retrospective cohort, *n* = 937)	Moderate	Internal	Moderate
Li et al., 2020 [30]	Retrospective High (small cohort, *n* = 63)	High	Internal	Limited
Koyama et al., 2024 [31]	Moderate (retrospective cohort, *n* = 459)	Moderate	Internal	Moderate

Qualitative risk-of-bias assessment of included studies based on four methodological domains: patient selection, risk of overfitting or data leakage, validation strategy, and clinical applicability. The assessment was inspired by principles from QUADAS-2 and PROBAST but adapted to accommodate the heterogeneous study designs included in this review. N/A: Not applicable.

## Data Availability

No new datasets were generated in this study. All data analyzed are derived from published studies included in the review and cited in the reference list.

## Supplementary Materials

The following supporting information can be downloaded at: https://www.mdpi.com/article/10.3390/cancers18060973/s1. Table S1: PRISMA checklist.

## Abbreviations

The following abbreviations are used in this manuscript:

NSCLC	Non-small-cell lung cancer
ALK	Anaplastic lymphoma kinase
TKIs	Tyrosine kinase inhibitors
AI	Artificial intelligence
ML	Machine learning
DL	Deep learning
CT	Computed tomography
PET-CT	Positron emission tomography/computed tomography
IHC	Immunohistochemistry
AUC	Area under the curve
Vs.	versus
EV	External validation
ANN	Artificial neural network
DAVID	Database for annotation, visualization, and integrated discovery
MUC5B	Mucin 5B
SFTPD	Surfactant protein D
DMBT1	Deleted in malignant brain tumors 1
SFTPA2	Surfactant protein A2
TFF3	Trefoil factor 3
CNN	Convolutional neural network
mRMR	Maximum relevance minimum redundancy
LASSO	Least absolute shrinkage and selection operator
HR	Hazard ratio
PFS	Progression-free survival
RSF	Random survival forest
CPH	Cox proportional hazard
